# 

**Published:** 1910-08

**Authors:** 


### CONTENTS.

ORIGINAL COMMUNICATIONS—
The Diagnosis and Treatment of Fractures. By Frank K. Boland, M. D., Atlanta, Georgia .... 217
The Public and Venereal Diseases. By R. H. Stanley, M. S., M. D., Albany, Ga. .. 221
Syphilis and the Paretic Group. By Stewart R. Roberts, M. D., Atlanta, Ga. .... 226
Development and Nerve Supply of the Intestinal Canal Surgically Considered. By J. L. Campbell, M. D., Atlanta, Ga. .... 230
Tuberculosis of Bones and Joints. By Theodore Toepel, M. D., Atlanta, Ga. .... 239
The Relation the House Fly Bears to Typhoid Fever and Other Infectious Diseases. By Dr. J. W. Palmer, Ailey, Ga. .... 244
Amylic and Ethylic Alcohol Differentiated; Physiological Action and Therapeutical Uses and Abuses of the Alcohol Group; Education as Essential as Legislation in Solving the Alcohol Problem. By B. W. Hall, A. B., M. D., Bowman, Ga. .... 251
EDITORIALS .... 257
NEWS AND NOTES .... 261
BOOK REVIEWS .... 263

